# Is perceived intolerance to milk and wheat associated with the corresponding IgG and IgA food antibodies? A cross sectional study in subjects with morbid obesity and gastrointestinal symptoms

**DOI:** 10.1186/s12876-018-0750-x

**Published:** 2018-01-30

**Authors:** Anne Stine Kvehaugen, Dag Tveiten, Per G. Farup

**Affiliations:** 1 Department of Surgery, Innlandet Hospital Trust, Kyrre Greppsgate 11, 2819 Gjøvik, Norway; 2 Lab1 AS, Sandvika, Norway; 3 Department of Research, Innlandet Hospital Trust, Brumunddal, Norway; 4 Unit for Applied Clinical Research, Department of Clinical and Molecular Medicine, Faculty of Medicine and Health Sciences, Norwegian University of Science and Technology, Trondheim, Norway

**Keywords:** Antibodies, Immunoglobulin G, Immunoglobulin a, Milk, Wheat, Gastrointestinal, Hypersensitivity, Obesity

## Abstract

**Background:**

Serum IgG and IgA food antibodies have been used for dietary advice to subjects with gastrointestinal symptoms and perceived food intolerance, but the role of these antibodies in mediating intolerance is controversial. The present study investigated associations between perceived gastrointestinal intolerance to milk-or wheat and the corresponding s-IgG and s-IgA food antibodies in subjects with morbid obesity.

**Methods:**

Subjects with morbid obesity (BMI ≥ 40 kg/m^2^ or ≥35 kg/m^2^ with obesity-related complications) were included. Irritable Bowel Syndrome (IBS) was diagnosed based on the Rome III criteria. Severity of specific gastrointestinal symptoms were measured with the Gastrointestinal Symptom Rating Scale (GSRS)-IBS. S-IgG against cow’s milk, cheese, wheat and gluten, and s-IgA against casein and gliadin were measured.

**Results:**

Ninety-seven subjects (80 females) with mean age 45 (SD 8.4) years were included, 70 had gastrointestinal complaints, 25 had IBS, and 22 and 20 reported milk- and wheat- intolerance respectively. There were no significant differences in serum concentrations or proportions of subjects above defined cut-off values for the antibodies between subjects with and without gastrointestinal complaints. In the group with gastrointestinal complaints, no significant differences were found between subjects with and without perceived food intolerance. Except for a significant correlation between IgG against cheese and GSRS-diarrhea (*Rho*: -0.25, *P* = 0.04), no significant correlations were found between the antibodies and type or degree of gastrointestinal symptoms, including IBS.

**Conclusions:**

The study showed no associations between perceived milk or wheat intolerance and the corresponding s-IgG and s-IgA food antibodies in subjects with morbid obesity.

## Background

Food-induced gastrointestinal (GI) symptoms are frequently reported in the population and associated with functional gastrointestinal disorders, in particular irritable bowel syndrome (IBS) [1]. Milk and dairy products, as well as wheat and/or gluten containing foods are among the foods often considered as triggers [2, 3]. Controversy still remains regarding the role of IgG food antibodies in mediating food intolerance. The measurement of serum concentrations of these antibodies has been and is still used by some laboratories as a guidance for dietary interventions [4]. Expert societies and guidelines on food allergy state that the evidence for the use of these tests is lacking; it has been proposed that the presence of IgG food antibodies merely reflects exposure to the corresponding foods and indicates immunological tolerance [4–7]. This view has been challenged by showing that IgG-guided elimination diets improved symptoms in different patient groups, including subjects with IBS and IBS with migraine [8–11]. However, there is a lack of studies investigating associations between perceived intolerance against particular foods and the corresponding s-IgG or s-IgA food antibodies in adults with GI symptoms. The present study addressed this issue in subjects with morbid obesity, a patient group with reportedly high prevalence of gastrointestinal symptoms, including IBS [12–14]. Interestingly, IBS and obesity also have several pathophysiological features in common, including increased gut permeability [15, 16], which could potentially result in increased translocation of food antibodies.

Aim of the present study was to explore the association between perceived intolerance to milk/dairy products and wheat and the serum concentrations (including cut-off values) of IgG and IgA against the corresponding food antigens in subjects with morbid obesity reporting GI complaints. Secondary aims were to investigate associations between 1) perceived intolerance to the foods in question; and 2) the IgGs and IgAs directed against these foods, with the following parameters; dietary intake of the offending foods, IBS, severity of specific GI symptoms and gut permeability.

## Methods

### Patients and study design

This cross-sectional study was performed at the unit for morbid obesity at Innlandet Hospital Trust, Gjøvik, Norway. Consecutive subjects referred for morbid obesity (BMI ≥ 40 kg/m^2^ or BMI ≥ 35 kg/m^2^ with obesity related complications (e.g. diabetes, hypertension, sleep apnoea, musculoskeletal problems) were included in a comprehensive study from December 2012 to September 2014. The patients were evaluated for bariatric surgery or non-surgical treatment of obesity, but had not started the weight reducing intervention at the time of study inclusion. A medical history was taken, a physical examination was performed, and a blood sample was collected for further analyses. The patients filled in paper-based questionnaires, including a case-report form and a food frequency questionnaire (FFQ). Subjects with major organic GI disorders (including celiac disease) or former major GI surgery, subjects who did not complete the FFQ or lacked blood samples for the biobank were excluded.

### Case-report form

included questions on a large range of variables (e.g.demographics, previous or present co-morbidities (diabetes mellitus, polycystic ovary syndrome, hypertension, cardiovascular disease, gallstones, hypo−/ hyperthyroidism, fibromyalgia, osteoporosis, chronic bronchitis, hay-fever, asthma, psychological problems, GI diseases), different health scores, perception of overall state of health, GI symptoms (yes/no) and food-induced GI symptoms). IBS was diagnosed with a validated Norwegian translation of the Rome III criteria [17]; and the degree of specific GI complaints with the Gastrointestinal Symptom Rating Scale – IBS (GSRS-IBS) [18]. The GSRS-IBS questionnaire contained 13 questions with responses ranging from 1 to 7 (no discomfort at all to very severe discomfort). The questions were clustered into the following dimensions; GSRS-diarrhea, −constipation, −bloating, −pain and –satiety, and a mean value for the items in each dimension were calculated. Questions regarding food-induced GI symptoms were recorded with our own questionnaire, with focus on milk/dairy products and wheat. This questionnaire included questions regarding whether or not the patients related their GI symptoms to the intake of these foods, as well as visual analogue scales (range 0–100) asking *“how certain are you that; Milk/dairy products are the offending foods? Wheat is the offending food?”*

For convenience, further mention of intolerance to milk and dairy in the text will be mentioned as intolerance to milk only.

### FFQ

The FFQ, designed to study the usual diet, has been prepared and validated by the University of Oslo [19, 20]. Daily intake of food, nutrients and energy was calculated by Department of Nutrition at the University of Oslo by their in-house calculation program (KBS, version 7.3, food database AE-14). The food composition database in the calculation program is based on the official Norwegian food composition table from 2016 [21] and is supplemented with additional food items.

### Laboratory analyses

Analyses of food antibodies were performed by Lab1, Sandvika in accordance with the manufacturer’s instructions. Serum zonulin, a marker of tight junction gut permeability (reference value < 48 ng/ml), and IgA against casein and gliadin were analyzed with Enzyme Linked Immunosorbent Assays (Zonulin ELISA Kit, Immundiagnostik AG, Bensheim, Germany, and; IgA gliadin/casein Screening ELISA test from Immunolab GmbH, Kassel, Germany). IgG antibodies against a panel of 44 common foods were measured with an enzyme immunoassay from R-Biopharm AG, Darmstadt, Germany (RIDASCREEN® Spec. IgG Foodscreen). From this panel of antibodies, only those related to the food intolerances in question were selected for statistical analyses. Four IgG food antibodies were considered relevant: IgG against cow’s milk, cheese, wheat and gluten. The following cut-off values for the food antibodies, as recommended by the manufacturers, were defined as test positive: For IgG a value ≥ 7.5 μg/ml was considered mildly elevated and a value ≥ 20 μg/ml was considered strongly elevated. The corresponding cut-off values for IgA were ≥ 8.0 U/ml (mildly elevated) and ≥ 12 U/ml (strongly elevated). Values below the lowest detection limit for the IgG food antibodies (2.5 μg/ml) were assigned the value 2.4 μg/ml.

### Statistical analyses

Data analyses were performed with IBM SPSS Statistics for Windows, Version 23 (IBM Corp., Armonk, N.Y., USA). For continuous data, comparisons between two groups were analyzed with the independent sample t-test if data were normally distributed or the non-parametric Mann Whitney U-test if data were skewed. Categorical data were analyzed with χ^2^ statistics. Correlations between variables were tested with Spearman’s correlation. Associations between perceived food intolerance and the corresponding food antibodies were adjusted for co-variates by use of logistic regression models (milk- or wheat intolerance as the dependent variable). A *P*-value < 0.05 was considered statistically significant.

### Sample size and power calculations

No power-calculation was performed as we were not aware of previously published studies with a similar study population and design.

## Results

In all, 97 out of 159 subjects were eligible for the present study (female/male: 80/17; mean age: 45 (SD 8.4) years). Of these, 70 had GI complaints. There were no significant differences between the groups with and without GI complaints regarding demographics, co-morbidity, blood tests, dietary intake or food antibodies (data not shown). All the presented analyses were based on the group with GI complaints. Twenty-two (31.4%) and 20 (28.6%) subjects related their symptoms to the intake of milk and wheat respectively; 11 (15.7%) had a perceived intolerance to both milk and wheat.

Table 1 shows the patient characteristics, biochemical and dietary data in subjects with and without perceived intolerance to milk or wheat. The presence of co-morbidities was not significantly different between those with and without perceived intolerance to either milk or wheat (data not displayed; all *P*-values > 0.05). The exception was IBS (Table 1) and hypothyroidism. The latter was significantly more common among subjects with than without perceived intolerance to wheat (5/18 (27.8%) vs. 2/48 (4.2%), *P* = 0.014). Alcohol consumption and smoking did not differ between those with and without milk or wheat intolerance (data not displayed; *P*-values > 0.05 for both).

**Table 1:** Patient characteristics, biochemical and dietary data in subjects with and without perceived milk-or wheat intolerance

	Perceived milk intolerance		*P*-value	Perceived wheat intolerance		*P*-value
	Yes (*n* = 22)	No (*n* = 48)		Yes (*n* = 20)	No (*n* = 50)	
Age, years	43.0 (7.39)	45.5 (8.91)	0.256 ^d^	46.3 (8.05)	44.1 (8.65)	0.312^d^
Gender (Female), n (%)	19 (86.4)	38 (79.2)	0.742 ^a^	18 (90.0)	39 (78.0)	0.322^a^
BMI, kg/m^2^	42.3 (2.70)	41.4 (3.39)	0.233 ^d^	42.0 (2.72)	41.5 (3.40)	0.631^d^
IBS (Rome III), n (%)	12/21 (57.1)	13/47 (27.7)	0.020 ^b^ *	10/18 (55.6)	15/50 (30.0)	0.054^b^
GSRS-IBS (range: 1–7)						
GSRS-Pain	2.50 (1.00–5.00)	1.50 (1.00–4.00)	0.014 ^c^ *	2.25 (1.00–5.00)	2.00 (1.00–4.00)	0.158^c^
GSRS-Bloating	2.33 (1.00–5.33)	2.50 (1.00–5.67)	0.493 ^c^	2.67 (1.00–5.33)	2.33 (1.00–5.67)	0.337^c^
GSRS-Diarrhea	1.75 (1.00–4.75)	1.50 (1.00–4.75)	0.065 ^c^	2.12 (1.00–4.75)	1.50 (1.00–4.75)	0.075^c^
GSRS-Constipation	1.00 (1.00–4.50)	1.00 (1.00–4.00)	0.916 ^c^	1.00 (1.00–4.50)	1.50 (1.00–4.00)	0.746^c^
GSRS-Satiety	1.00 (1.00–4.00)	1.50 (1.00–4.50)	0.669 ^c^	1.25 (1.00–4.50)	1.50 (1.00–4.00)	0.493^c^
S-Zonulin ≥48 ng/ml, n (%)	16 (72.7)	29 (60.4)	0.318 ^b^	9 (45.0%)	36 (72.0)	0.033^b^*
Dietary intake, g/day						
Milk, cream and cheese	238 (7.00–1173)	469 (23.4–1801)	0.053 ^c^	NA	NA	NA
Bread, cereals and cakes	NA	NA	NA	274 (26.4–734)	280 (138–707)	0.654^c^
How certain are you that milk/wheat is the offending food? (VAS-scale: 0–100; a high value indicates high degree of certainty)	77.5 (13.0–100)	NA	NA	67.0 (17.7–95.8)	NA	NA

Data are given as mean (SD), median (min-max) or n (%)

*IBS* Irritable Bowel Syndrome, *GSRS-IBS* Gastrointestinal Symptom Rating Scale-IBS, *NA* Not applicable, *VAS-scale* Visual Analogue Scale

*Statistically significant: *P* < 0.05

^a^Fisher’s Exact Test

^b^Pearson Chi-Square

^c^Mann-Whitney U test

^d^Independent sample t-test

Tables 2 and 3 show the serum concentrations of the relevant food antibodies in subjects with and without perceived intolerance to milk and wheat respectively, with comparisons between the groups, and the number and proportion of subjects with food intolerance in the groups with positive and negative tests for the given cut-off values. Comparisons between the groups in question did not reveal any significant differences (Table 2 and Table 3). The results remained non-significant after adjusting for the intake of the offending foods (data not shown, all *P*-values > 0.05). For wheat intolerance versus no intolerance, additional adjustments for differences in s-Zonulin ≥48 ng/ml (as shown in Table 1) and hypothyroidism did also not alter the non-significant relationship between the antibodies and the reported intolerance (*P*-values > 0.05).

**Table 2:** Associations between the s-IgG and s-IgA milk antibodies and perceived milk intolerance

	Perceived milk intolerance		*P*-value †	Cut-off ǂ	Perceived milk intolerance (22/70 (31.4))		*P*-value ‡
	Yes (*n* = 22)	No (*n* = 48)			Test positive	Test negative	
IgG total (μg/ml) (*n* = 69)	9.77 (2.19)	10.6 (2.08)	0.143				
IgG cow’s milk (μg/ml)	15.1 (2.40–112)	10.9 (2.40–117)	0.713	≥ 7.5 ≥ 20	14/43 (32.6) 9/23 (39.1)	8/27 (29.6) 13/47 (27.7)	0.797^b^ 0.332^b^
IgG cheese (μg/ml)	5.99 (2.40–72.0)	3.84 (2.40–88.9)	0.755	≥ 7.5 ≥ 20	8/23 (34.8) 3/14 (21.4)	14/47 (29.8) 19/56 (33.9)	0.672^b^ 0.524^a^
IgA casein (U/ml)	5.05 (2.70–65.0)	5.15 (2.10–33.1)	0.556	≥ 8.0 ≥ 12	7/25 (28.0) 4/12 (33.3)	15/45 (33.3) 18/58 (31.0)	0.645^b^ 1.000^a^

†Data are given as mean (SD) or median (min-max) and independent sample t-test or Mann-Whitney-U-test is performed

ǂPositive test results as defined by cut-off values recommended by the manufacturer

‡Data are given as proportion (percent) and *X*^*2*^-statistics is performed

^a^Fisher’s Exact Test

^b^Pearson Chi-Square

**Table 3:** Associations between the s-IgG and s-IgA wheat antibodies and perceived wheat intolerance

	Perceived wheat intolerance		*P*-value †	Cut-off ǂ	Perceived wheat intolerance (20/70 (28.6))		*P*-value ‡
	Yes (n = 20)	No (n = 50)			Test positive	Test negative	
IgG total (μg/ml) (*n* = 69)	10.3 (2.03)	10.3 (2.19)	0.939				
IgG wheat (μg/ml)	8.30 (3.23–84.6)	8.93 (2.40–126)	0.933	≥ 7.5 ≥ 20	12/41 (29.3) 5/13 (38.5)	8/29 (27.6) 15/57 (26.3)	0.878^b^ 0.498^a^
IgG gluten (μg/ml)	11.6 (5.77–82.4)	11.4 (2.40–139)	0.459	≥ 7.5 ≥ 20	17/52 (32.7) 6/19 (31.6)	3/18 (16.7) 14/51 (27.5)	0.195^b^ 0.734^b^
IgA gliadin (U/ml) (*n* = 69)	5.40 (3.40–8.70)	4.70 (3.00–15.9)	0.337	≥ 8.0 ≥ 12	3/7 (42.9) 0/1 (0)	16/62 (25.8) 19/68 (27.9)	0.384^b^ 1.000^a^

†Data are given as mean (SD) or median (min-max) and independent sample t-test or Mann-Whitney-U-test is performed

ǂPositive test results as defined by cut-off values recommended by the manufacturer

‡Data are given as proportion (percent) and *X*^*2*^-statistics is performed

^a^Fisher’s Exact Test

^b^Pearson Chi-Square

Table 4 shows the correlations between the antibodies investigated and the variables addressed in Table 1. Additionally, because hypothyroidism was significantly more common among subjects with than without perceived intolerance to wheat, correlations between the antibodies and hypothyroidism were examined as post-hoc analyses. Results are included in Table 4.

**Table 4:** Correlations between the food antibodies and the patient characteristics, biochemical and dietary data

	IgG cow’s milk	IgG cheese	IgA casein	IgG wheat	IgG gluten	IgA gliadin
Age, years	Rho: 0.05, *P* = 0.67	Rho: 0.11, *P* = 0.35	Rho: −0.04, *P* = 0.73	Rho: 0.09, *P* = 0.44	Rho: 0.03, *P* = 0.82	Rho: −0.02, *P* = 0.90
Gender (0 = female, 1 = male)	Rho: 0.14, *P* = 0.24	Rho: 0.20, *P* = 0.09	Rho: 0.14, *P* = 0.26	Rho: 0.04, *P* = 0.72	Rho: 0.03, *P* = 0.79	Rho: 0.15, *P* = 0.22
BMI, kg/m^2^	Rho: 0.08, *P* = 0.53	Rho: −0.02, *P* = 0.87	Rho: 0.17, *P* = 0.15	Rho: 0.03, *P* = 0.81	Rho: 0.09, *P* = 0.46	Rho: 0.12, *P* = 0.33
IBS (0 = no, 1 = yes)	Rho: 0.04, *P* = 0.74	Rho: −0.03, *P* = 0.82	Rho: 0.04, *P* = 0.74	Rho: −0.16, *P* = 0.20	Rho: − 0.04, *P* = 0.72	Rho: 0.06, *P* = 0.62
GSRS-Pain (range: 1–7)	Rho: −0.04, P = 0.73	Rho: − 0.09, *P* = 0.47	Rho: − 0.10, *P* = 0.41	Rho: − 0.07, *P* = 0.57	Rho: 0.10, *P* = 0.40	Rho: 0.07, *P* = 0.59
GSRS-Bloating (range: 1–7)	Rho: −0.11, *P* = 0.36	Rho: − 0.08, *P* = 0.53	Rho: − 0.04, *P* = 0.72	Rho: − 0.14, *P* = 0.25	Rho: − 0.02, *P* = 0.85	Rho: − 0.08, *P* = 0.52
GSRS- Diarrhea (range: 1–7)	Rho: − 0.18, *P* = 0.14	Rho: − 0.25, *P* = 0.04*	Rho: −0.19, *P* = 0.13	Rho: − 0.11, *P* = 0.35	Rho: 0.01, *P* = 0.91	Rho: 0.05, *P* = 0.66
GSRS-Constipation (range: 1–7)	Rho: 0.06, *P* = 0.61	Rho: 0.05, *P* = 0.70	Rho: 0.06, *P* = 0.60	Rho: −0.07, *P* = 0.55	Rho: −0.05, *P* = 0.70	Rho: 0.03, *P* = 0.80
GSRS-Satiety (range: 1–7)	Rho: <−0.01, *P* = 0.98	Rho: −0.05, *P* = 0.70	Rho: − 0.20, *P* = 0.10	Rho: 0.02, *P* = 0.87	Rho: 0.11, *P* = 0.36	Rho: −0.20, *P* = 0.09
S-Zonulin ≥48 ng/ml	Rho: − 0.02, *P* = 0.89	Rho: 0.05, *P* = 0.69	Rho: 0.03, *P* = 0.80	Rho: −0.10, *P* = 0.40	Rho: − 0.10, *P* = 0.41	Rho: 0.27, *P* = 0.02*
Hypothyroidism (0 = no, 1 = yes)	Rho: 0.06, *P* = 0.63	Rho: 0.09, *P* = 0.47	Rho: 0.14, *P* = 0.25	Rho: 0.26, *P* = 0.04*	Rho: 0.22, *P* = 0.07	Rho: 0.15, *P* = 0.23
Dietary intake, g/day						
Milk, cream and cheese	Rho: 0.01, *P* = 0.94	Rho: −0.02, *P* = 0.84	Rho: − 0.06, *P* = 0.61	NA	NA	NA
Bread, cereals and cakes	NA	NA	NA	Rho: −0.12, *P* = 0.30	Rho: −0.09, P = 0.44	Rho: 0.13, *P* = 0.27
How certain are you that milk/wheat is the offending food? (VAS-scale: 0–100; a high value indicates high degree of certainty) †	Rho: 0.30, *P* = 0.18	Rho: 0.19, *P* = 0.41	Rho: 0.09, *P* = 0.68	Rho: 0.05, *P* = 0.83	Rho: −0.14, P = 0.57	Rho: −0.30, *P* = 0.23

*NA* Not applicable, *IBS* Irritable Bowel Syndrome, *GSRS* Gastrointestinal Symptom Rating Scale-IBS, *VAS-scale* Visual Analogue Scale

†Included only those with perceived milk or wheat intolerance respectively

*Statistically significant: *P* < 0.05

## Discussion

There is a lack of studies investigating the association between patient reported food intolerances and the corresponding IgG or IgA food antibodies in adults with GI symptoms. The novelty of the present study is that IgG and IgA antibodies directed against milk and wheat were compared between subjects who all reported GI complaints, but who did or did not perceive themselves as intolerant to these foods. Dietary intake of the offending foods was also estimated, as dietary restrictions have been suggested to affect serum concentrations of the antibodies in some studies [22–24]. Milk/dairy consumption was lower and close to the level of statistical significance in those with than without perceived milk intolerance. No differences in the intake of wheat containing foods were seen between those with and without perceived wheat intolerance. The measured food antibodies did however not discriminate between those with and without perceived food intolerances, either in unadjusted analyses, or adjusted for the intake of the offending foods. We think that our inclusion of symptomatic subjects only, represents the subjects likely to seek medical/nutritional advice and undergo antibody testing. On the other hand, initial analyses did not reveal any differences between those with and without GI complaints either, regardless of perceived intolerance to milk or wheat.

We are aware of only a few previous studies with a similar design as the present study. One is a study from our group which demonstrated poor correspondence between perceived intolerance against gluten and milk/cheese in patients with IBS and common tests for food intolerance, including IgG and IgA against gliadin and gluten, and IgA against lactalbumin, lactoglobulin and casein [3]. Another is a study by Anthoni et al., which opposed to the present study detected slightly higher titers of IgG against milk protein in subjects with than without self-reported milk-induced GI symptoms [22]. However, likewise to the present study, no significant positive associations were found between the IgG antibodies and either IBS or specific GI symptoms [22].

Other studies on the relationship between IBS and IgG food antibody levels have brought mixed results, but correlations between the antibodies and type or severity of symptoms were rarely demonstrated, or even inverse correlations were found [23, 25, 26]. This is in contrast to intervention studies showing that exclusion diets guided by elevated IgG serum concentrations improved symptoms in patients with IBS [8–11]. However, these studies were associated with various limitations, such as the lack of blinding and/or the lack of a control group, incomplete details about how the diets were designed, or intervention and control diets being too different. Importantly, none of the studies compared the effect of excluding the same foods in those with high versus low serum concentrations of the corresponding food antibodies. We speculate that the divergent findings, with the lack of correlations with symptoms in this and most other cross-sectional studies on the one hand, and intervention trials showing symptomatic improvements on the other hand, may be due to the many potential triggering components of the same food item. With respect to milk and wheat, several components, separate or in combination, may provoke symptoms (e.g. different proteins, peptides, lactose and fructans) [27–29]. Lactose and fructans both belongs to the Fermentable Oligo-Di-Monosaccharides And Polyols (FODMAPs), which collectively have been shown to play a role in IBS [30]. In open, uncontrolled trials, the placebo effect can also not be excluded. The lack of standardized methods in IgG testing is also a problem that may have affected differences between studies.

Increased gut permeability could potentially result in elevated levels of the food antibodies. Increased gut permeability, reflected by s-zonulin (a marker of tight junction permeability in the gut) [31], did not predict either serum concentrations of the food antibodies or perceived food intolerance in the present study. An exception was IgA against gliadin, which correlated positively with increased levels of s-zonulin. In spite of this, and in line with a previous study [32], there was an inverse association between tight-junctional gut permeability and perceived wheat intolerance, suggesting other underlying mechanisms for the reported intolerance.

Although not among our main study hypotheses, the present study showed that hypothyroidism was more common among subjects with than without perceived intolerance to wheat. Hypothyroidism also correlated significantly with IgG against wheat, and marginally with IgG against gluten. Numerous reports have described a link between celiac disease and thyroid disease [33, 34], but associations between non-celiac gluten−/wheat sensitivity and autoimmune diseases, such as autoimmune thyroiditis, remain elusive [32, 35–37]. Although not specific for the condition, anti-gliadin antibodies, in particular of the IgG sub-class, has been reported as a frequent serological marker in non-celiac gluten sensitivity in several studies [32, 36, 37]. Much controversy still remains regarding the clinical entity of “non-celiac wheat−/non-celiac gluten sensitivity” [38]. Nevertheless, the present results, in light of previous findings, may deserve closer attention in future studies.

### Strengths and limitations

It was a strength that the study was performed pragmatically in a routine clinical setting in conformity with daily clinical practice, and that food intolerance was based on perceived GI intolerance. The antibody tests have commonly been used in this way. The design, therefore, increased the external validity. Food intolerance based on double-blind placebo-controlled food provocation, which is considered the gold standard in diagnosing adverse food reactions [39], could have given other results.

Another strength was the clearly formulated questions asking specifically about food-induced GI symptoms and not about other adverse food reactions, then followed by separate questions regarding the relation to milk or wheat. The degrees of certainty of the perceived associations between the food item and the GI complaints were also assessed.

Patients with celiac disease were excluded from the study. If systematic testing and exclusion of patients with IgE-mediated food allergy and lactose intolerance had also been performed, this could have yielded different results. Based on European data, the prevalence of IgE sensitization in adults were found to be approximately 2% [40] to 4.5% [41] for wheat and 0.8% for milk [41], suggesting a minor influence of these conditions to the present results. The prevalence of lactose malabsorption in Norway is also low, approximating 4% both in the general population and in patients with IBS [42]. Eliminating a potential confounding effect of FODMAPs would require the use of a FODMAP restricted elimination and provocation diet and was not performed. To use the tests for food antibodies only after thorough examinations is however not according to everyday use of the tests.

Another consideration is that the study was accomplished in subjects with morbid obesity referred for weight-reducing interventions. The literature suggests that overweight and obese subjects have a high frequency of GI co-morbidity, including IBS [12–14]. We therefore considered this study population as suitable for the present study question. If the patients had been recruited primarily due to GI complaints and with more severe symptoms, the results might have been different.

The many statistical tests performed increased the risk of a type 1 statistical error. Inaccuracies in the dietary reports, including the risk of re-call bias, can also not be excluded. Moreover, the dietary data did not contain exact information about the amount consumed of the different grains/flour types, such as wheat; only surrogate markers such as bread, cakes and cereals which often contain wheat. These aspects could perhaps explain the lack of a correlation between the intake of the offending foods and the corresponding food antibodies in this study. The significance of the amount consumed of particular foods in increasing the serum concentrations of the corresponding food antibodies seems however inconclusive [22–24, 43].

## Conclusion

The study showed no associations between perceived milk and wheat intolerance and the corresponding IgG and IgA food antibodies and gave no support for the use of these antibodies for dietary advice to an unselected group of subjects with morbid obesity and GI complaints.

## Abbreviations

ELISA: Enzyme Linked Immunosorbant Assay
FFQ: Food Frequency Questionnaire
FODMAPs: Fermentable Oligo-Di-Monosaccharides And Polyols
GI: Gastrointestinal
GSRS-IBS: Gastrointestinal Symptom Rating Scale – IBS
IBS: Irritable Bowel Syndrome
Ig: Immunoglobulin
